# S-CDK-regulated bipartite interaction of Mcm10 with MCM is essential for DNA replication

**DOI:** 10.3389/fcell.2024.1420033

**Published:** 2024-09-19

**Authors:** Xueting Wang, Lu Liu, Mengke Chen, Yun Quan, Jiaxin Zhang, Huiqiang Lou, Yisui Xia, Hongxiang Chen, Wenya Hou

**Affiliations:** ^1^ Department of Dermatology, Huazhong University of Science and Technology Union Shenzhen Hospital, Shenzhen, China; ^2^ Guangdong Key Laboratory for Biomedical Measurements and Ultrasound Imaging, Nation Regional Key Technology Engineering Laboratory for Medical Ultrasound, School of Biomedical Engineering, Shenzhen University Medical School, Shenzhen, China; ^3^ Guangdong Key Laboratory for Genome Stability and Disease Prevention, Shenzhen University General Hospital and Medical School, Shenzhen, China

**Keywords:** cell cycle, DNA replication, Mcm10, phosphorylation, protein–protein interaction

## Abstract

Mcm10 plays an essential role in the activation of replicative helicase CMG through the cell cycle-regulated interaction with the prototype MCM double hexamer in *Saccharomyces cerevisiae*. In this study, we reported that Mcm10 is phosphorylated by S-phase cyclin-dependent kinases (S-CDKs) at S66, which enhances Mcm10–-MCM association during the S phase. S66A single mutation or even deletion of whole N-terminus (a.a. 1–128) only causes mild growth defects. Nevertheless, S66 becomes indispensable in the absence of the Mcm10 C-terminus ((a.a. 463–571), the major MCM-binding domain. Using a two-degron strategy to efficiently deplete Mcm10, we show that *mcm10*-S66AΔC has a severe defect in proceeding into the S phase. Notably, both lethality and S-phase deficiency can be rescued by artificially tethering *mcm10*-S66AΔC to MCM. These findings illustrate how the Mcm10–MCM association is regulated as a crucial event in DNA replication initiation.

## Introduction

DNA replication, the faithful duplication of genetic material, is a fundamental process crucial for the propagation of life. This intricate mechanism involves the coordinated action of numerous proteins orchestrating the unwinding of the double helix, the synthesis of new DNA strands, and the faithful transmission of genetic information to daughter cells (O’Donnell et al., 2013). Among the plethora of proteins involved, Mcm10 stands out for its indispensable but less understood role in the complex choreography of DNA replication. The significance of Mcm10 lies in its ability to act as a versatile coordinator, regulating various stages of DNA replication, including initiation (Douglas et al., 2018; Langston et al., 2023; Henrikus et al., 2024), progression (Langston et al., 2017; Looke et al., 2017), and termination (Campos et al., 2023), as well as replication-coupled nucleosome assembly (Zhao et al., 2023).

Mcm10 is among the minimal set of essential firing factors for reconstituted DNA synthesis *in vitro* (Yeeles et al., 2015), and it has been demonstrated to be crucial for the maturation of the helicase complex, which unwinds the DNA double helix, allowing the initiation of DNA synthesis in *Saccharomyces cerevisiae* (Kanke et al., 2012; van Deursen et al., 2012; Watase et al., 2012). Mcm10 binds multiple subunits of the Cdc45-MCM-GINS (CMG) holo-helicase, which is formed by the six Mcm2–7 ATPases, the Cdc45 protein, and the GINS complex (Bell and Labib, 2016; Costa and Diffley, 2022), thereby stimulating its helicase activity or bypassing blocks on lagging strand DNA during replication elongation (Langston et al., 2017; Looke et al., 2017). Mcm10 can also promote the progression of stalled forks, including those under conditions of topological stress during replication termination (Campos et al., 2023). On the other hand, Mcm10 also possesses strand annealing activity that can prevent fork regression caused by enzymes triggering fork reversal (Mayle et al., 2019).

During replication initiation, Mcm10 facilitates the remodeling of the MCM double hexamers (DHs) and subsequently triggers the activation of the assembled CMG helicase (Quan et al., 2015; Douglas et al., 2018; Lewis et al., 2022; Langston et al., 2023; Henrikus et al., 2024). Mcm10 specifically binds MCM DHs loaded on the chromatin through an intricate mode involving at least the N-terminal and C-terminal domains of Mcm10 with multiple Mcm2–7 subunits (Quan et al., 2015; Douglas and Diffley, 2016; Liu et al., 2020). Mcm10 N-terminus contributes to low-affinity interaction with MCM, and the C-terminus mediates high-affinity interaction (Quan et al., 2015). Moreover, their interaction is cell cycle-regulated with a relatively weak “G1-like” and strong “S-like” mode, as demonstrated *in vivo* and *in vitro* (Quan et al., 2015; Douglas and Diffley, 2016). However, the mechanistic details of how the Mcm10–MCM DH interaction is regulated have yet to be defined.

In this study, we investigate the cell cycle-regulated Mcm10–MCM interaction and elucidate its critical role in DNA replication initiation. Through a combination of *in vitro* and *in vivo* biochemical approaches, we show that Mcm10 is a novel substrate of S-phase cyclin-dependent kinases (S-CDKs). The phosphorylation of a single conserved site (S66) within the Mcm10 N-terminus enhances its association with the MCM complex. Phospho-mutation S66A alone does not exert any apparent effect on cell growth under normal conditions. However, the loss of both Mcm10 Ser66 phosphorylation and the C-terminal MCM-binding domain causes cell death, underscoring the synergistic role of phosphorylation and protein–protein interaction. Moreover, by utilizing the two-degron strategy along with cell cycle synchronization, we efficiently deplete Mcm10 protein in the late G1 phase and observe a severe S-phase defect in mcm10-S66AΔC. Intriguingly, both lethality and S-phase deficiency can be rescued by artificially tethering Mcm10-S66AΔC to MCM. These data provide insights into the cell cycle-regulated bivalent Mcm10–MCM interaction by S-CDKs and its essential role in orchestrating DNA replication although neither the Mcm10 N-terminus- nor C-terminus-mediated interaction alone is indispensable.

## Results

### Mcm10 phosphorylation facilitates its association with MCM

**FIGURE 1:**
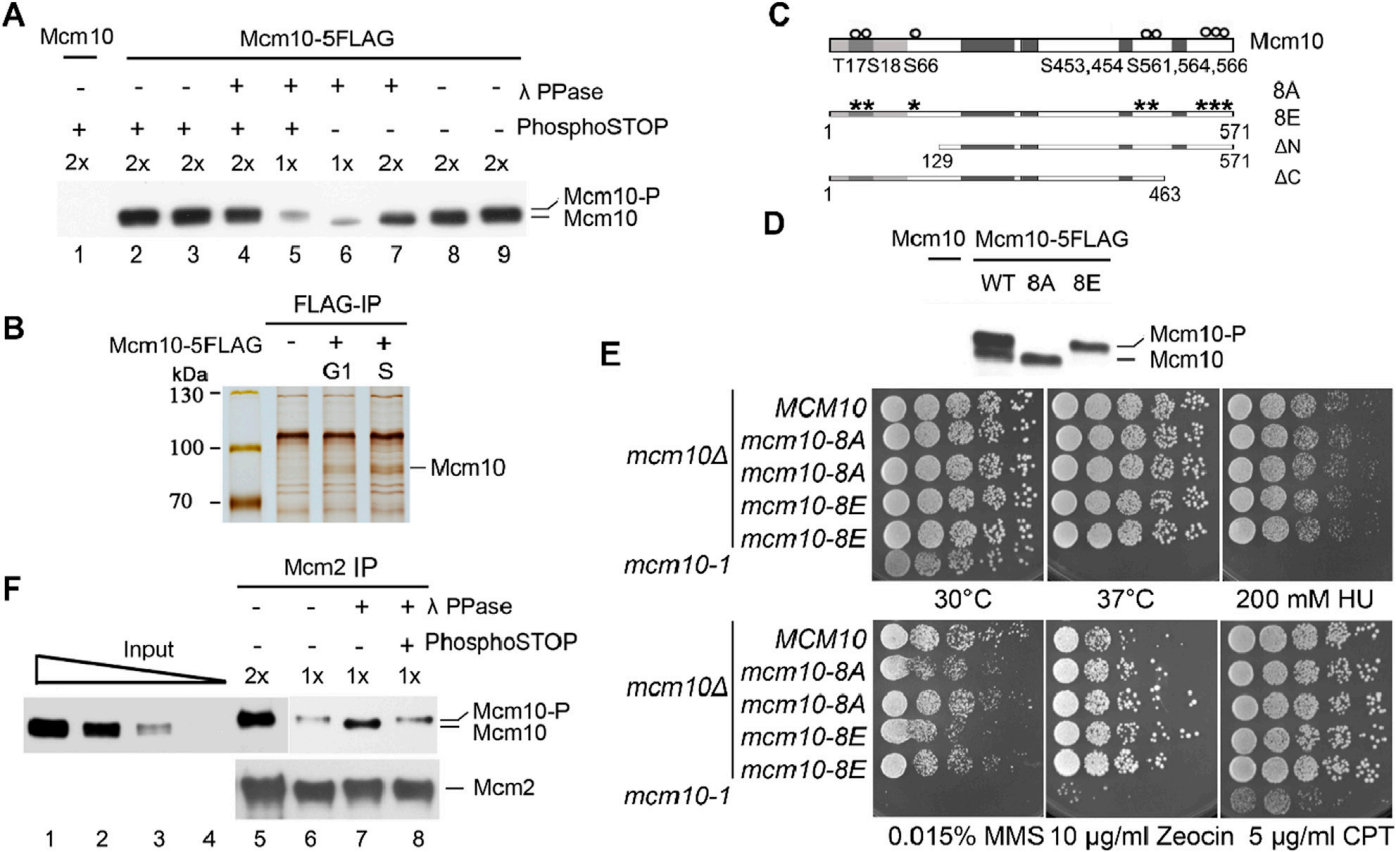
The phosphorylation of Mcm10 may facilitate its association with MCM. **(A)** Mcm10 undergoes phosphorylation *in vivo*. Yeast cells carrying 5FLAG-tagged Mcm10 (Strain QY317, Table 1) at its endogenous locus were grown to the exponential phase. Cell lysates were resolved by a high-resolution 8% PAGE containing SDS. Cells with untagged Mcm10 were applied as a control. λ phosphatase (PPase) or PPase inhibitor cocktails (PhosphoSTOP) were added to the lysates before SDS-PAGE. **(B, C)** Mapping of Mcm10 phosphorylation sites by immunoprecipitation-coupled mass spectrometry analysis (IP/MS). Yeast cells carrying 5FLAG-tagged Mcm10 (Strain QY317, Table 1) at its endogenous locus were cultured and arrested in G1 by α-factor. Cells were released for 0 min (G1) and 40 min (S) before collection. Cell lysates were incubated with anti-FLAG M2 affinity gel. Bound fractions were eluted by 0.5 μg/μL FLAG peptides and resolved by 8% PAGE containing SDS. Untagged Mcm10 was subjected to the same procedure as a control. The Mcm10 bands were excised and analyzed by MS/MS. **(C)** Putative phosphorylation sites. **(D)** Mutations of all eight putative phosphorylation sites to A or E abrogate the shift of Mcm10. The alanine (*mcm10*-8A) or glutamic acid (*mcm10*-8E) substitutions of all eight putative phosphorylation sites (S or T) are marked as asterisks. Darker boxes represent relatively conserved domains. **(E)** Phospho-mutants of *MCM10*, 8A, and 8E show nearly wild-type growth under normal or stressed conditions. *mcm10-8A/E* mutant strains were obtained by plasmid shuffling on plates containing 5-FOA. Wild-type *MCM10* was cloned and expressed in the pRS316 vector to allow the *mcm10*Δ mutant to grow. The pRS316-*MCM10* plasmid was removed from 5-FOA plates because its *URA3* expression converted 5-FOA into a toxin. Rescue plasmids expressing *MCM10* or its mutants were tested to examine their ability to support cell growth. Five-fold serial dilutions of log phase cells were spotted on the indicated plates and incubated for 2°days at 30°C, unless otherwise stated, before being photographed. **(F)** Phosphorylated Mcm10 may preferentially bind to Mcm2. Mcm2 was immunoprecipitated by anti-Mcm2 antibodies. The input and immunoprecipitation (IP) fractions were resolved by a high-resolution gel. The IP fractions were subjected to λ-PPase and/or PhosphoSTOP treatment to verify the phosphorylation form. The amounts loaded were titrated (1x and 2x) to achieve good separation.

In previous studies, we reported a crucial role of cell cycle-regulated interaction of Mcm10 with MCM DHs in the remodeling and activation of the latter (Quan et al., 2015; Liu et al., 2020). Mcm10 is recruited to origins via low-affinity (without CDK and DDK activity, G1-like) and high-affinity (with CDK and DDK activity, S-phase-like) modes, as demonstrated in the *in vitro* reconstitution system (Douglas and Diffley, 2016). However, how the Mcm10–MCM DH interaction is regulated remains unknown. We noticed that the Mcm10 protein occasionally displays two forms migrating very closely on immunoblots (Figure 1A). Since the two bands were so close, we postulated that the slower-migrating one might be a phosphorylated form of Mcm10. To test this, we treated the cell lysates with λ phosphatase (λ PPase) prior to separation on a high-resolution polyacrylamide gel. The relatively slower-migrating band disappeared after λ PPase treatment (Figure 1A, lane 7). This result became clearer when only half of the samples were loaded (lane 6). The sensitivity of this slower-migrating band to λ phosphatase was specific because it was retained if PPase inhibitor cocktails (PhosphoSTOP) were added simultaneously (compare lanes 4–7). These results reveal that Mcm10 may undergo phosphorylation *in vivo*.

To investigate the physiological role of Mcm10 phosphorylation, we first mapped post-translational modification sites on Mcm10. Endogenous Mcm10-5FLAG protein was precipitated from cells synchronized in G1 (by α-factor) or S phase (release for 40 min) (Figure 1B). Liquid chromatography–mass spectrometry/mass spectrometry (LC–MS/MS) revealed eight possible phosphorylation sites near the Mcm10 N-terminus (T17, S18, and S66 cluster) and C-terminus (S453, 454, 561, 564, and 566) (Figure 1C). To investigate the possible role of Mcm10 phosphorylation, we mutated all these sites to alanine (*mcm10*-8A) or glutamic acid (*mcm10*-8E). Since *MCM10* is essential for cell viability, the *mcm10* mutants were constructed via plasmid shuffling. WT *MCM10* was cloned and expressed on a pRS316/*URA3* single-copy vector to allow the growth of *mcm10*Δ. The mutant *mcm10* allele in a second vector, pRS313/*HIS3*, was also introduced. The pRS316-*MCM10* plasmid can be eliminated on 5-fluoroorotic acid (5-FOA) plates because it expresses *URA3*, which converts 5-FOA to a toxin. Thus, the growth on 5-FOA plates reflects the physiological function of the copy of the *mcm10* mutant expressed on pRS313. Five-fold serial dilution of log phase cells was spotted on SC-His plates in the presence or absence of 5-FOA. Mcm10-8A and mcm10-8E proteins migrated as non-phosphorylated and phosphorylated forms, respectively (Figure 1D). This indicates that the observed shift is due to phosphorylation among these sites. Both mutants grew as well as the wild-type (WT) regardless of being under normal or various stress conditions (Figure 1E), suggesting that Mcm10 phosphorylation has no apparent effect on overall cell growth under the tested conditions.

Next, we examined whether Mcm10 phosphorylation affects this interaction using co-immunoprecipitation (CoIP). Endogenous Mcm2 was precipitated with anti-Mcm2 antibodies. Phosphorylated Mcm10 seemed to be enriched in the Mcm2-bound fraction compared to the input samples (Figure 1F, lanes 6 and 7). To confirm this, we treated Mcm2 precipitates with λ PPase and observed a PPase inhibitor-sensitive shift of Mcm10 to the faster-migrating form (Figure 1F, compare lanes 7–9). These data suggest that phosphorylation may facilitate the Mcm10–MCM interaction *in vivo* although recombinant Mcm10 binds directly to Mcm2, as shown in the previous *in vitro* pull-down assays (Quan et al., 2015).

### The Mcm10 S66 phospho-mutant is synthetic lethal with its C-terminus truncation

**FIGURE 2:**
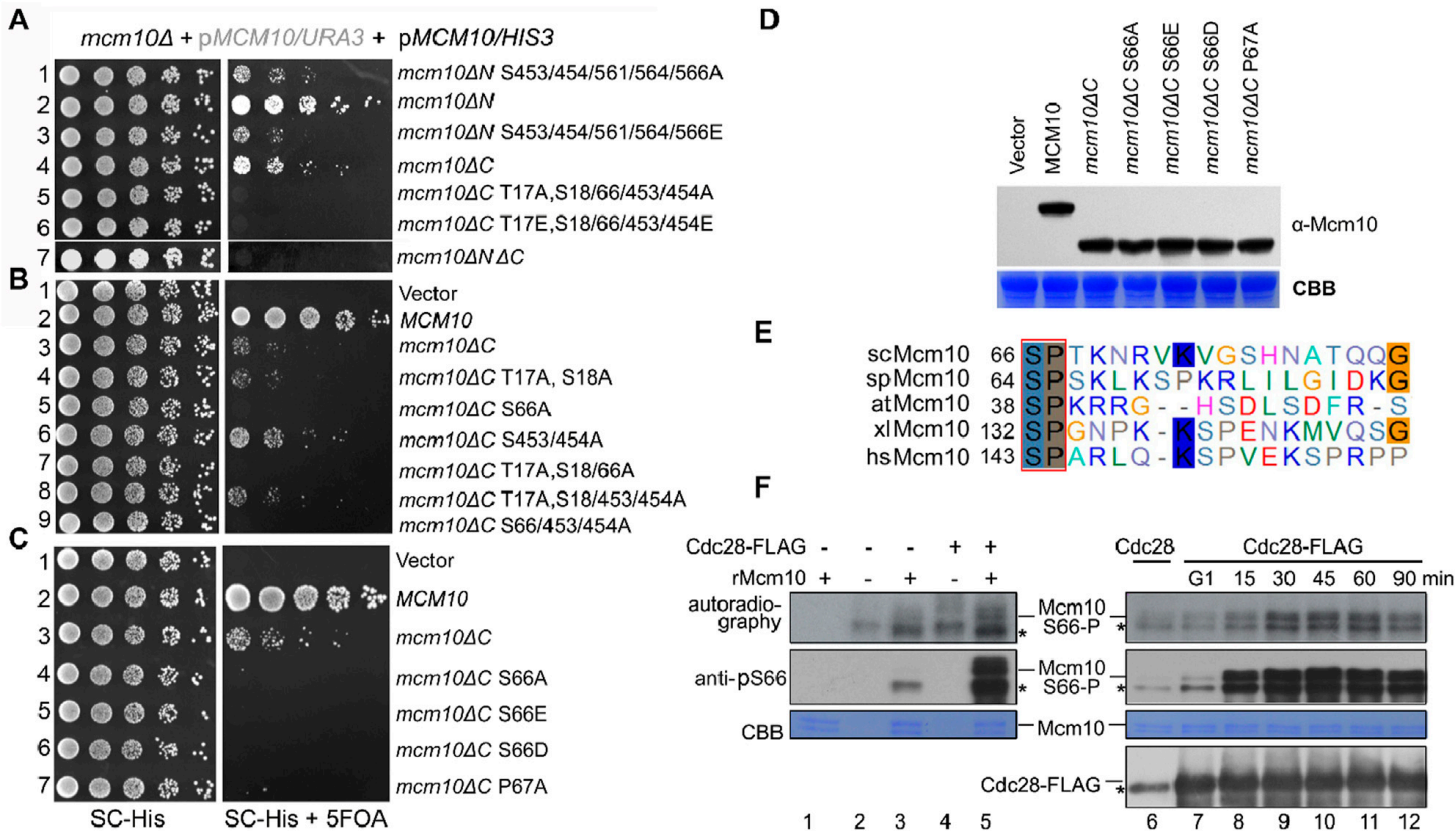
Mcm10 S66 is a substrate of S-CDKs and becomes indispensable in the absence of the Mcm10 C-terminus **(A)** *mcm10* phosphorylation mutants show a synergistic defect with loss of the N- or C-terminal interaction region. Plasmid shuffling and serial dilution analyses were conducted similarly to the methods shown in Figure 1E. **(B, C)** Mapping of the critical phosphorylation site(s) required for the viability of *mcm10*ΔC. S66, a putative CDK target, is the sole phosphorylation site required for the growth of *mcm10ΔC*. **(D)** All *mcm10*ΔC derivative inviable alleles are expressed at a comparable level to wild-type *MCM10*. Whole-cell extracts were prepared from log-phase cells and immunoblotted by an anti-FLAG antibody. The membrane was then stained with Coomassie brilliant blue (CBB) to show the exact loading. **(E)** The CDK target site in Mcm10 may be conserved from yeast to human. The amino acid sequence of Mcm10 from different organisms was aligned using ClustalW. **(F)** *In vitro* kinase assays. Cdc28-5FLAG was immunoprecipitated from asynchronized (left panel) or synchronized cells released from G1 arrest for the indicated time (right panel) and then incubated with purified recombinant Mcm10 proteins in the presence of γ-^32^P-ATP. Mcm10 phosphorylation was detected by autoradiography (top panel) or anti-pS66 immunoblots (middle panel). Nonspecific bands were labeled by “*”.

Phosphorylation sites were enriched in the Mcm10 N-terminus (Mcm10-N, a.a., 1–128) and C-terminus (Mcm10-C, a.a., 461–571), which mediate interactions with MCM. Such a multivalent interaction mode prompted us to examine the synthetic effect of these factors on cell growth. Correlating with the relatively greater contribution of Mcm10-C than Mcm10-N to interactions with MCM, the *mcm10*ΔC allele showed much weaker growth than *mcm10*ΔN (Figure 2A, lines 4 and 2). Interestingly, the deletion of both Mcm10-N and Mcm10-C resulted in minimal growth (line 7). We then combined phosphorylation mutations with either *mcm10*ΔN or *mcm10*ΔC. Mutating five C-terminal phosphorylation sites displayed synthetic sickness with *mcm10*ΔN (lines 1 and 3), whereas mutating three N-terminal sites in *mcm10*ΔC caused cell death (lines 5 and 6). These results indicate that phosphorylation is important for the proper function of Mcm10 *in vivo*. Next, we determined which phosphorylation site(s) are indispensable for the growth of *mcm10*ΔC. Through serial mutation analysis, we demonstrated that mutation of the S66 residue alone is synthetic lethal with *mcm10*ΔC (Figure 2B, lines 5, 7, and 9). Moreover, mimicking both non-phosphorylated (A) and phosphorylated (E or D) mutations displayed similar phenotypes. This may be explained by either of two possibilities. First, neither S66D nor S66E may function as phospho-mimics. Second, reversible phosphorylation of S66 is important for the survival of *mcm10*ΔC. The *mcm10*-S66ΔC mutants were expressed at a comparable level relative to WT *in vivo* in these experiments, thus excluding the possibility that the lethality could be due to a failure in the expression of *mcm10* alleles (Figure 2D). These data suggest that a single phosphorylation site in the Mcm10 N-terminus (S66) becomes indispensable in the absence of the C-terminus, the major MCM-interaction motif.

Interestingly, S66 is followed by a conserved canonical “P-X-K-X-R” motif recognized by CDKs (Figure 2E). Moreover, *mcm10*-P67AΔC phenocopied *mcm10*-S66AΔC (Figure 2C, line 7), implicating that Mcm10 S66 may be a substrate of CDKs. To test this, we first conducted a kinase assay using purified Cdc28-FLAG from asynchronized yeast cells. In the presence of [γ-^32^P]-ATP, purified recombinant Mcm10 was phosphorylated by Cdc28, as indicated by both autoradiography and immunoblots using antibodies specific to phosphorylated S66 (pS66) (Figure 2F, lane 5). To examine which CDKs phosphorylate Mcm10, we next purified Cdc28 kinase from cells after release from G1 and repeated the *in vitro* kinase assays. S66 phosphorylation was barely detectable by CDKs from G1 cells (Figure 2F, lanes 6–7) and significantly increased by CDKs from S cells (compare lanes 7–12). Meanwhile, all reactions contained similar amounts of Cdc28. These data suggest that Mcm10 S66 might be a target of S-CDKs.

**FIGURE 3:**
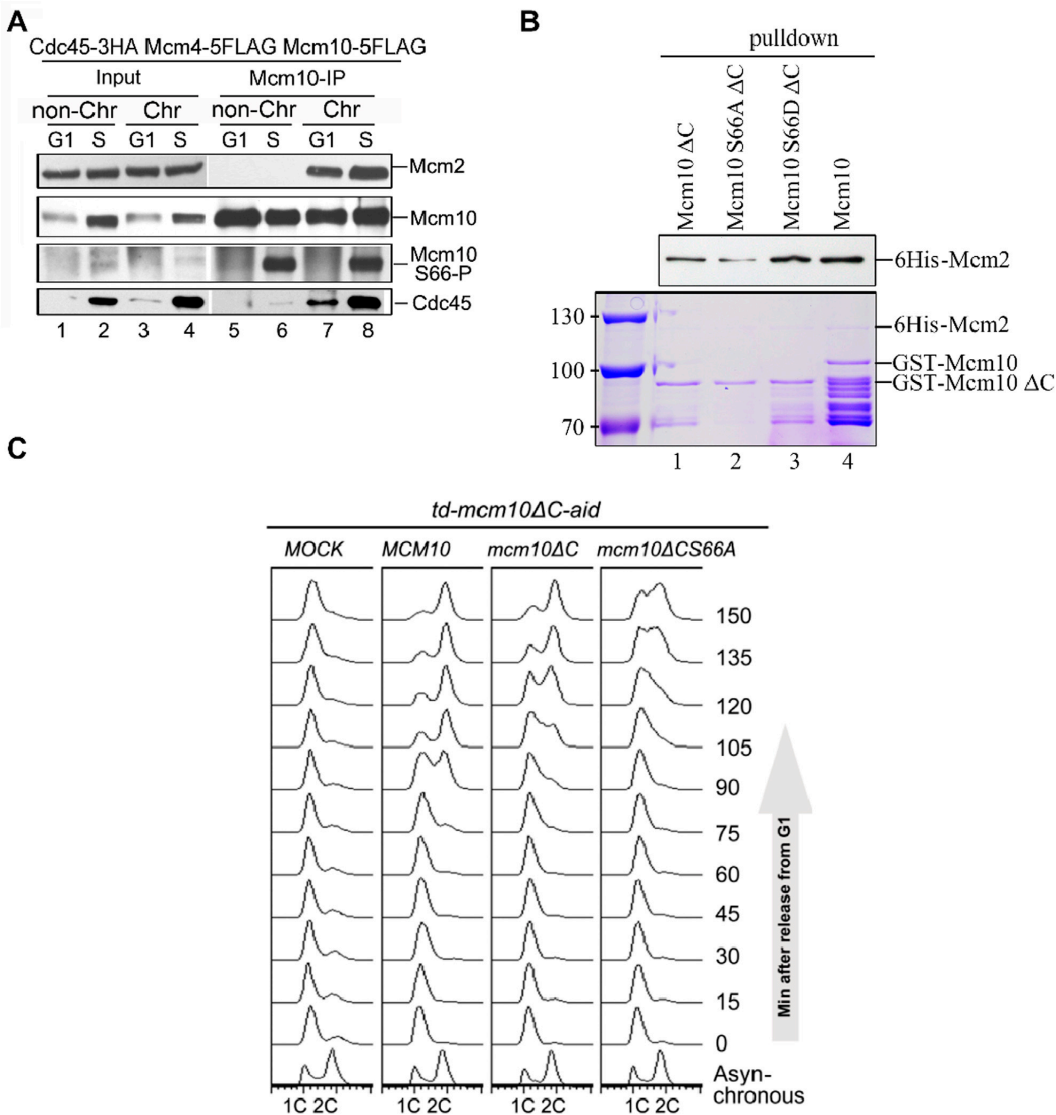
The Mcm10–Mcm2 interaction is an essential requirement of replication initiation. **(A)** Mcm10-pS66 occurs prior to being loaded onto chromatin in the S phase. Mcm10-FLAG was precipitated from either non-chromatin or chromatin-associated fractions and then subjected to Mcm10-IP with the anti-Mcm10 antibody. **(B)** Mcm10-S66 phospho-mutations affect the interaction with Mcm2 *in vitro*. GST pull-down assays were performed using affinity-purified 6-His-Mcm2 along with GST-tagged Mcm10 and its mutant proteins. The bound proteins were separated by SDS-PAGE and detected either by immunoblotting against anti-His antibodies or CBB staining. **(C)** *mcm10* mutants are defective in S-phase entry and progression. Endogenous Mcm10 protein was depleted by the two-degron strategy as above. The *td-mcm10*ΔC*-aid* cells bearing a serial of *mcm10* mutant plasmids were released from α-factor synchronization. The ability of each *mcm10* allele to support DNA synthesis was monitored by flow cytometry.

To validate these *in vitro* observations, we next probed Mcm10 S66 phosphorylation during the cell cycle *in vivo*. After collecting synchronized G1- and S-phase (release from α-factor for 60 min) cells, we prepared spheroplasts and fractionated soluble proteins into non-chromatin-bound (non-Chr) and chromatin-bound (Chr) through a sucrose cushion, as described previously (Quan et al., 2015; Liu et al., 2020). Both fractions were subjected to Mcm10-IP. The protein level of Cdc45, which is heavily increased upon S-phase entry, can be used as an indicator of the cell cycle stage (Figure 3A, lanes 1–2). In agreement with previous results (Quan et al., 2015), both Mcm2 and Cdc45 co-precipitated with Mcm10 exclusively in Chr fraction (Figure 3A, compare lanes 7–8 with 5–6), demonstrating a successful chromatin fractionation. However, as shown by anti-pS66 immunoblotting (IB), Mcm10-S66 phosphorylation was clearly detected in both non-Chr and Chr fractions (Figure 3A, compare lanes 6 and 8). This indicates that S66 phosphorylation occurs prior to Mcm10 recruitment to chromatin. In terms of timing, S66 was phosphorylated only in S cells but not in G1 cells (Figure 3A, compare lanes 5 and 6). Consistent with *in vitro* kinase activities shown in Figure 2F, these data confirm that Mcm10 is a *bona fide* substrate of S-CDKs.

Coinciding with the appearance of S66 phosphorylation, the Mcm10–Mcm2 interaction was relatively increased during the S phase (Figure 3A, compare lanes 7 and 8), which is reminiscent of the preferential binding of phosphorylated Mcm10 with Mcm2, as shown in Figure 1F. To test whether S66 phosphorylation contributes to the enhanced Mcm10–Mcm2 association, we conducted pulldown assays using affinity-purified recombinant proteins. As shown in Figure 3B, Mcm10-S66D∆C displayed a relatively stronger Mcm2-binding than Mcm10-S66A∆C and Mcm10-∆C (compare lanes 1–3). These *in vivo* and *in vitro* biochemical data suggest that although the Mcm10–MCM interaction is mainly mediated by the Mcm10 C-terminus, it can be enhanced by the phosphorylation of S66 at its N-terminus during the S phase. Together with the genetic results shown in Figure 2, which show that loss of both interaction motifs (*mcm10*-S66A∆C) causes cell death, we postulated that the lethality of *mcm10*-S66AΔC may be due to the essential function of Mcm10 in DNA replication. To test it, we measured cell cycle progression by flow cytometry. Since the *mcm10*-S66AΔC mutant is lethal, we tested its ability to support DNA synthesis in the Mcm10 conditional knockout background. Since very small residual Mcm10 can support its essential function, we used a previously developed two-degron system, temperature-induced degron (*td*) and auxin-induced degron (*aid*), in the *mcm10*ΔC allele, which had been shown to deplete endogenous Mcm10 protein efficiently. Under this condition, the DNA content did not change after release from G1 for over 150 min (Figure 3C, Mock). Next, we introduced a set of *mcm10* alleles to test whether they could support DNA synthesis after depletion. DNA increased more slowly to 2C content in *mcm10*ΔC than in WT (Figure 3C, 90–120 min). For *mcm10*-S66AΔC cells, the S phase started approximately 30 min later than *mcm10*ΔC and failed to reach 2C even after 150 min. These data indicate that the lethality of *mcm10*-S66AΔC is likely due to a failure in DNA synthesis. These data provide genetic evidence that MCM-binding mediated by both Mcm10 C-terminus and S66 phosphorylation defines an indispensable role of Mcm10 in replication.

### The restoration of the Mcm10–CMG interaction rescues the lethality and replication defects in *mcm10*Δ*C-S66A*

**FIGURE 4:**
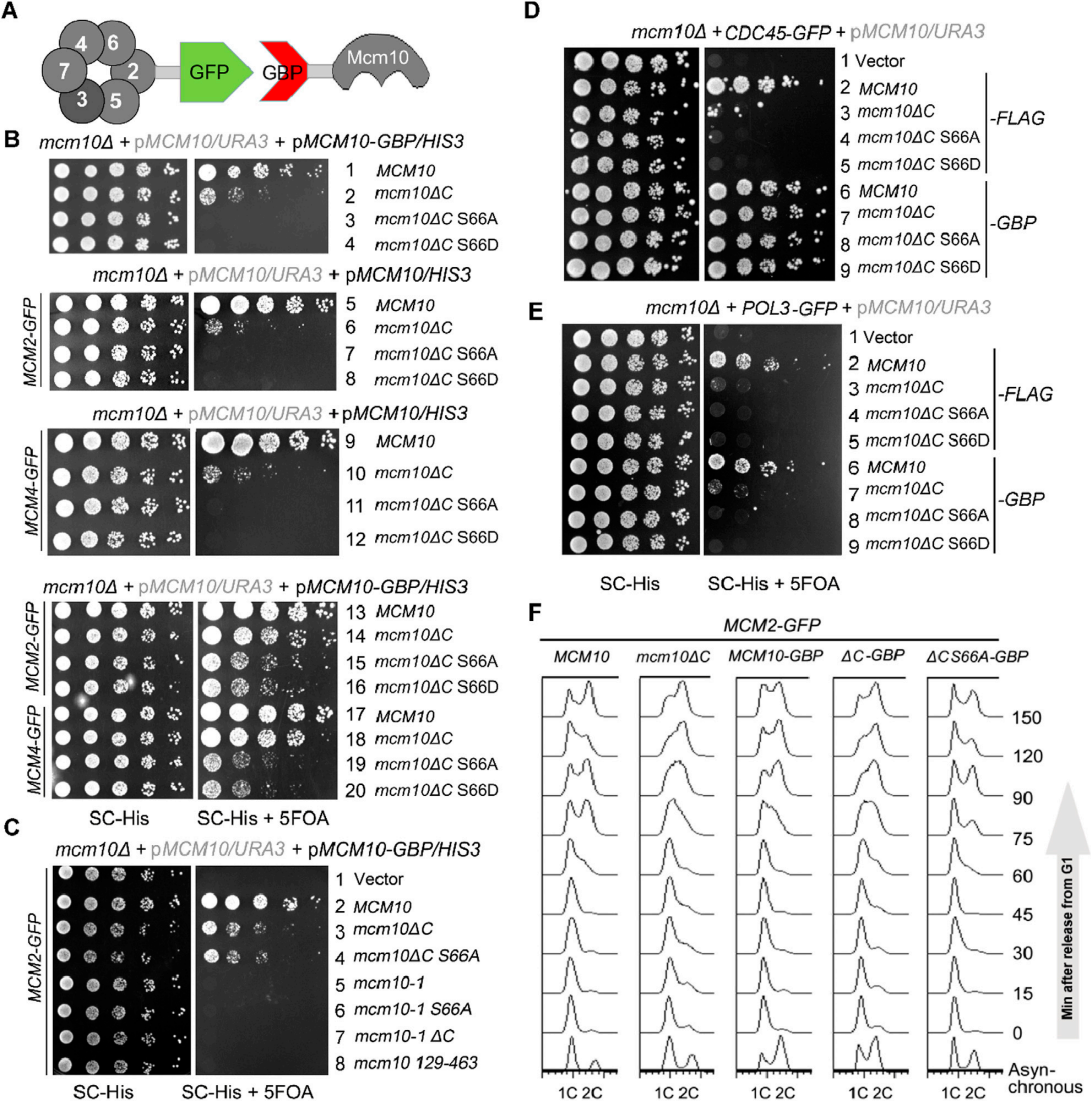
The lethality of *mcm10ΔC-S66* mutants can be suppressed by restoring their interaction with either Mcm2 or Mcm4. **(A)** An *in vivo* GFP trap strategy to enforce the Mcm10–MCM interaction. Mcm2 or Mcm4 was tagged with GFP, while Mcm10 or its mutant form was fused with GBP. Interactions between any two proteins might be restored artificially via the GFP–GBP pair through an *in vivo* GFP trap experiment. **(B, C)** *In vivo* GFP trap of Mcm10 with either Mcm2 or Mcm4 suppresses the lethality of *mcm10*-S66ΔC alleles. Either Mcm10-GBP or Mcm2-GFP with a single tag showed no effect on the growth of wild-type or *mcm10* alleles. Plasmid shuffling and serial dilution assays were carried out, as shown in Figure 2. **(D, E)** *In vivo* GFP trap of Mcm10 with either Cdc45 **(D)** or Pol2 **(E)**. Mcm10 mutants carrying a 5FLAG tag were applied as negative controls, which showed no effect on the growth in either Cdc45-GFP or Pol3-GFP cells. Plasmid shuffling and serial dilution assays were carried out, as shown above. **(F)** *In vivo* GFP trap of Mcm10 with Mcm2 partially also suppresses the S-phase defect of *mcm10*-S66ΔC alleles. Cells were synchronized at G1 with the α-factor and released into the S phase. Cell cycle progression was monitored by flow cytometry analysis of the DNA content for each *mcm10*ΔC allele.

If the failure/delay in S-phase entry is solely caused by the compromised Mcm10–MCM interaction in *mcm10*-S66AΔC, it should be suppressed by reinforcing this interaction. We adopted an *in vivo* GFP trap strategy to achieve this (Figure 4A). If we add a GFP tag to one protein and a GBP (GFP binding protein) tag to another protein, these two proteins can be tethered to each other through strong affinity between the GFP and GBP pair. In Figure 4B, we introduced a pRS313/*HIS3* plasmid expressing each *mcm10* allele with or without a GBP tag at the C-terminus by plasmid shuffling. Control experiments showed that Mcm10-GBP or Mcm2-GFP supported WT growth (Figure 4B, lines 1 and 5). The co-expression of Mcm10-GBP and Mcm2-GFP also displayed normal growth (Figure 4B, line 13), indicating that the dissociation of Mcm10 and Mcm2 is not important for normal growth. Notably, strains expressing *mcm10*-S66AΔC-GBP or *mcm10*-S66DΔC-GBP became viable depending on the presence of Mcm2-GFP (compare lines 3, 4, 7, 8, 15, and 16). However, *mcm10* (129–463) (deleting both N- and C-terminus) and the temperature-sensitive allele *mcm10-1* could not be rescued by fusing with Mcm2 (Figure 4C, compare lines 4–8). These results suggest that the lethality of *mcm10*-S66ΔC is very likely due to the loss of its association with Mcm2. Since multiple Mcm2–7 subunits are partners of Mcm10, we also checked whether tethering the interaction of the defective mutant of Mcm10 with other partners has a similar effect as Mcm2. As shown side by side in Figure 4B, *mcm10*-S66ΔC-GBP survived in the presence of Mcm4-GFP and Mcm2-GFP. Moreover, tethering *mcm10*-S66ΔC to Cdc45, a subunit of the CMG complex, could rescue their lethality as well (Figure 4D). However, tethering Mcm10 to Pol3 had no rescue effect at all (Figure 4E). Pol3 is a subunit of DNA Pol δ, which is involved in replication progression but not in initiation. Therefore, we propose that the lethality of these *mcm10* mutations is attributable to defective interactions with MCM or its activator Cdc45 during replication initiation. Furthermore, flow cytometry profiles showed that the *mcm10*-S66AΔC mutant could gradually proceed into the S phase and reach 2C content dependent on fusion with Mcm2 (Figure 4F). These results suggest that the restoration of Mcm10–CMG interactions can suppress the lethality and replication deficiency of *mcm10*-S66ΔC. Hence, we propose that S-CDK-regulated Mcm10 N-terminus-mediated interaction, along with Mcm10 C-terminus-mediated interaction with MCM, defines an indispensable step in DNA replication initiation in budding yeast.

## Discussion

CMG maturation is characterized by a cascade of events: origin DNA melting (Lewis et al., 2022), dissociation of the transient dimeric CMG (Quan et al., 2015; Liu et al., 2020), and exclusion of the lagging strand from the central channel of the CMG ring (Henrikus et al., 2024). Mcm10 is instrumental in facilitating these structural transitions (Yeeles et al., 2015; Douglas et al., 2018). Moreover, Mcm10 has been demonstrated to stimulate the helicase activity of the recombinant CMG complex *in vitro* (Langston et al., 2017; Langston and O’Donnell, 2019; Langston et al., 2023) and induce CMG remodeling under stress (Wasserman et al., 2019), suggesting its direct effect on the CMG complex. Our results lead us to posit that the interaction between Mcm10–MCM/CMG is a critical determinant for the orchestration of CMG remodeling, particularly throughout the CMG maturation during initiation. Subsequent investigations might explore whether S-CDK-regulated Mcm10–MCM association is involved in the complete separation of the splayed dimeric CMG, especially considering that Mcm10 binds to the N-terminal domains of Mcm2, Mcm4, and Mcm6 (Quan et al., 2015; Douglas and Diffley, 2016), which are posited as the interfaces of MCM double hexamers (Li et al., 2015). Additionally, phosphorylation of MCM10 has been reported in human cells (Izumi et al., 2000) and *Xenopus* (Chadha et al., 2016). With the recent paradigm shift in the understanding of initiation mechanisms in metazoa (Cvetkovic et al., 2023; Lim et al., 2023; Xia et al., 2023; Terui et al., 2024), further studies are warranted to elucidate whether the Mcm10–MCM interaction is subject to cell cycle-dependent regulation in higher eukaryotes.

## Experimental procedures

### Strains and plasmids

The strains and plasmids used in this study are listed in Tables 1, 2, respectively. Mutants were generated using recombination-mediated cassette exchange or tetrad dissection, as previously described (Quan et al., 2015). All the constructs were confirmed by DNA sequencing.

**TABLE 1:** Strains used in this study.

Strain	*Genotype*	Source
BY4741	*MATahis3Δ1 leu2Δ0 met15Δ0 ura3Δ0*	Gift from Dr. Junbiao Dai
BY4742	*MATα his3Δ1 leu2Δ0 lys2Δ0 ura3Δ0*	Gift from Dr. Junbiao Dai
W303-1a	*MAT*a *trp1-1 ura3-1 his3-11*,*15 leu2-3*,*112 ade2-1 can1-100 RAD5*	Gift from Dr. Judith L. Campbell
QY317	*W303 MATa KanMX6::MCM10-5FLAG*	This study
QY336	*W303 HIS3::MCM2-3HA KanMX6::MCM10-5FLAG*	This study
QY606	*BY4741 MATa his3Δ1 leu2Δ0 met15Δ0 ura3Δ0 lys2Δ0 mcm10Δ::KanMX6 pMCM10/URA3*	This study
QY713	*BY4741 MATa his3Δ1 leu2Δ0 met15Δ0 ura3Δ0 lys2Δ0 mcm10Δ::KanMX6 pMCM10/URA3 LEU2::MCM2-GFP*	This study
QY715	*BY4741 MATa his3Δ1 leu2Δ0 met15Δ0 ura3Δ0 lys2Δ0 mcm10Δ::KanMX6 pMCM10/URA3 LEU2::MCM4-GFP*	This study
QY6129	*BY4741 MATa his3Δ1 leu2Δ0 met15Δ0 ura3Δ0 lys2Δ0 mcm10Δ::KanMX6 pMCM10/HIS3 LEU2::MCM4-5FLAG NatMX::CDC45-3HA*	This study
QY6131	*BY4741 MATa his3Δ1 leu2Δ0 met15Δ0 ura3Δ0 lys2Δ0 mcm10Δ::KanMX6 pmcm10ΔC/HIS3 LEU2::MCM4-5FLAG NatMX::CDC45-3HA*	This study
QY6141	*BY4741 MATa his3Δ1 leu2Δ0 met15Δ0 ura3Δ0 lys2Δ0 mcm10Δ::KanMX6 pMCM10/HIS3 LEU2::MCM4-5FLAG NatMX::CDC45-3HA (p317MCM2-GFP::LYS2)*	This study
QY6142	*BY4741 MATa his3Δ1 leu2Δ0 met15Δ0 ura3Δ0 lys2Δ0 mcm10Δ::KanMX6 pmcm10ΔC/HIS3 LEU2::MCM4-5FLAG NatMX::CDC45-3HA (p317MCM2-GFP::LYS2)*	This study
QY394	*BY4741 KanMX::td-mcm10(1–463)-aid ubr1::P*_*GAL1*_ *-UBR1-P*_*GAL1*_ *-OsTIR1-9MYC-URA3*	This study

**TABLE 2:** Plasmids used in this study.

Plasmid	Base plasmid/genotype	Source
pET28a-MCM10	*kan* ^*r*^ *6His- MCM10*	This study
pGEX-4T-1-MCM10	*amp* ^*r*^ *GST- MCM10*	This study
pGEX-4T-1-*mcm10(1–128)*	*amp* ^*r*^ *GST- mcm10 (1–128)*	This study
pGEX-4T-1-*mcm10(1–463)*	*amp* ^*r*^ *GST- mcm10 (1–463)*	This study
pGEX-4T-1-*mcm6(1–439)*	*amp* ^*r*^ *GST- mcm6 (1–439)*	This study
pGEX-4T-1-*mcm4(1–471)*	*amp* ^*r*^ *GST- mcm4 (1–471)*	This study
pGEX-4T-1-*mcm10(464–571)*	*amp* ^*r*^ *GST- mcm10 (464–571)*	This study
pGEX-6P-1-MCM10	*amp* ^*r*^ *GST- MCM10*	This study
pRS313-MCM10-5FLAG	*amp* ^*r*^ */HIS3 MCM10-5FLAG*	This study
pRS313-*mcm10 ΔN*	*amp* ^*r*^ */HIS3 mcm10 Δ(1–128) -5FLAG*	This study
pRS313-*mcm10 ΔC*	*amp* ^*r*^ */HIS3 mcm10 Δ(464–571) -5FLAG*	This study
pRS313-*mcm10 S66A*	*amp* ^*r*^ */HIS3 mcm10-S66A-5FLAG*	This study
pRS313-*mcm10 P67A*	*amp* ^*r*^ */HIS3 mcm10-P67A-5FLAG*	This study
pRS313-*mcm10 ΔC S66A*	*amp* ^*r*^ */HIS3 mcm10 Δ(464–571) S66A-5FLAG*	This study
pRS313-*mcm10 ΔC S66E*	*amp* ^*r*^ */HIS3 mcm10 Δ(464–571) S66E-5FLAG*	This study
pRS313-*mcm10 ΔC S66D*	*amp* ^*r*^ */HIS3 mcm10 Δ(464–571) S66D-5FLAG*	This study
pRS313-*mcm10 ΔC P67A*	*amp* ^*r*^ */HIS3 mcm10 Δ(464–571) P67A-5FLAG*	This study
pRS313-MCM10-GBP	*amp* ^*r*^ */HIS3 MCM10-GBP*	This study
pRS313-*mcm10 ΔC* -GBP	*amp* ^*r*^ */HIS3 mcm10 Δ(464–571)-GBP*	This study
pRS313-*mcm10 ΔC S66A*-GBP	*amp* ^*r*^ */HIS3 mcm10 Δ(464–571)S66A-GBP*	This study
pRS313-*mcm10 ΔC S66D*-GBP	*amp* ^*r*^ */HIS3 mcm10 Δ(464–571)S66D-GBP*	This study
pRS317-MCM2-GFP	*amp* ^*r*^ */LYS2 MCM2-GFP*	This study

### Cell synchronization and flow cytometry analysis

A total of 7.5 μg/mL of α factor was added for cell synchronization in the G1 phase. G1 arrested cells were released by filter washing twice in a fresh medium and continued growth for the indicated time. Samples were collected and fixed with 70% ethanol and then processed for flow cytometry using a BD Biosciences FACSVerse machine.

### Whole-cell extracts and immunoblotting

Whole-cell extracts (WCEs) of 100 OD600 units of asynchronized or synchronized cells were prepared by glass bead beating (Mini-Beadbeater-16, BioSpec, United States) in lysis buffer [45 mM HEPES, pH 7.2, 150 mM NaCl, 1 mM EDTA, 10% glycerol, 0.2% NP-40, 1 mM PMSF, 2 mM DTT, 1× Protease Inhibitor Cocktail tablet (Roche), and 1× PhosSTOP tablet (Roche)]. Protein fractions were separated by SDS polyacrylamide gel electrophoresis (PAGE) and transferred to a PVDF membrane. Each protein was probed with the antibody specifically indicated in each figure by Western blotting. The antibodies used in this study are anti-Cdc45 (gift from Dr. Karim Labib), mouse anti-FLAG M2-specific monoclonal antibody (1:2000, Sigma), mouse anti-HA 16B12 (1:1000, Millipore), polyclonal anti-GST (glutathione transferase) (1:1000, OriGene), anti-6-His antibodies (1:1000, OriGene), anti-tubulin (1:10,000, MBL), and anti-Rad53 (1:1000, Abcam); protein-G-agarose (GE Healthcare) and NHS-activated agarose resins (GE Healthcare) were also used. HRP-conjugated anti-rabbit or anti-mouse IgG was used as the secondary antibody (1:10,000, Sigma). Anti-pS66 (Mcm10 S66 phosphorylation) was developed in rabbits against oligopeptides IEVPQ{pS}PTKNRVKVC (GenScript), {pS} stands for the phosphorylated Ser residue (GenScript).

### Phosphatase treatment

Mcm10-5FLAG in whole-cell extracts or co-immunoprecipitated with Mcm2 was treated with 200 U λ phosphatase (New England Biolabs) in the presence or absence of PhosphoSTOP (Roche) at 30°C for 20 min.

### Immunoprecipitation

Immunoprecipitation (IP) analysis was performed using strains co-expressing the tagged versions of each protein at a physiological level, as indicated in each figure. IP was carried out as described previously. Input (IN) corresponding to approximately 100 μg total protein was analyzed in parallel with immunoprecipitates. Proteins were analyzed by mass spectrometry or Western blotting using the indicated antibodies.

### Affinity purification and mass spectrometry

Mcm10-5FLAG was precipitated from whole-cell extracts using M2 affinity gel (Sigma). Nonspecific bound proteins were removed by washing with 0.5 μg/μL FLAG peptide. The bound fraction was boiled in an equal volume of 2x SDS loading buffer and resolved on an 8% SDS-PAGE and silver staining. An untagged strain was subjected to the same procedure as a control. The bands specific to the Mcm10-5FLAG sample were cut and digested by trypsin (NEB), followed by mass spectrometry analysis (Q Exactive™ Hybrid Quadrupole-Orbitrap Mass Spectrometer, Thermo Fisher Scientific).

### Protein expression and purification

Full-length and truncated forms of pGEX4T-1-MCM10, pGEX-4T-1-*mcm6(1–439)*, pGEX-4T-1-*mcm4(1–471)*, pET28a-MCM10, pET28a-MCM2, and pET28a-*mcm2(1–299)* constructs used in the biochemical experiments were expressed in *E. coli* BL21 (DE3) RIL codon-plus (Stratagene) and purified using affinity tags, followed by conventional column chromatography.

### Preparation of antibodies and Mcm10 agarose beads

To produce polyclonal antibodies specific to Mcm10 or Mcm2, the purified full-length protein was used to immunize rabbits. Polyclonal antibodies were affinity-purified. Mcm10 beads were prepared by immobilizing purified Mcm10 protein to NHS-activated agarose beads, as recommended by the manufacturer (GE Healthcare), which was used for an efficient *in vitro* pull-down assay.

## Preparation of antibodies

Antibodies specific to Mcm10 S66 phosphorylation were developed in rabbits against oligopeptides IEVPQ{pS}PTKNRVKVC. Antibodies were affinity-purified using non-phosphorylated oligopeptides to remove the antibody reacting with the non-phosphorylated polypeptide (GenScript).

### *In vitro* pull-down assay

Approximately 10 pmol of each protein was mixed with glutathione-Sepharose 4B (GE Healthcare Life Sciences), or anti-Mcm10 agarose beads made in this study were mixed in 100 μL of the binding buffer (50 mM HEPES-NaOH, pH 7.6, 150 mM NaCl, 10% glycerol, 1 mM EDTA, 1 mM PMSF, 1 μg/μL BSA, and 0.1% Triton X-100) and incubated for 1 h at 4°C. The beads were washed at least three times prior to Western blotting and/or Coomassie staining.

### *In vitro* kinase assay

Cdc28-5FLAG from WCE of 50 OD600 units of asynchronized or G1-released cells was precipitated with 10 μL M2 affinity gel (Sigma) and washed five times with lysis buffer and once with kinase buffer [20 mM HEPES/KOH, pH 7.2, 80 mM β-glycerophosphate, 10 mM MgCl, 20 mM EGTA, 100 μM ATP, protease inhibitor tablets (EDTA free, Roche), and PhosphoSTOP (Roche)]. Each reaction mixture (20 μL) contained 10 μL Cdc28-5Flag-bound beads, 2 μg GST-Mcm10, and 2 μ*Ci* γ-^32^P-ATP. After incubation at 30°C for 30 min, the reaction products were separated by 8% SDS-PAGE and transferred onto a PVDF membrane.

### Construction of *mcm10* alleles

The viability of various *mcm10* alleles was determined by plasmid shuffling since *MCM10* is an essential gene. Wild-type *MCM10* was cloned and expressed in the pRS316/*URA3* vector to allow *mcm10Δ* mutants to grow. The pRS316-*MCM10* plasmid was removed on 5-FOA plates because it expresses *URA3*, which caused cells toxic to 5-FOA. The ability to support cell growth was tested for various *mcm10* alleles expressed in the pRS313/*HIS3* vector under a range of genetic backgrounds, as indicated in each figure. Five-fold serial dilution of log phase cells was spotted on SC-His plates in the presence or absence of 5-FOA and incubated for 2 days at the indicated temperature before photography.

### *In vivo* GFP trap assay (protein tethering assay)

The interaction between *mcm10* mutants and Mcm2–7 was restored through a tethering strategy using the *in vivo* GFP trap (Quan et al., 2015). In Mcm10–Mcm2–7 tethering experiments, each *mcm10* allele was fused to GBP, while the Mcm2 or Mcm4 subunit was tagged with GFP. To ensure specifically targeted protein tethering, the omission of one of the GFP/GBP pairs was included as controls in all tethering assays.

## Data Availability

The original contributions presented in the study are included in the article/supplementary material, further inquiries can be directed to the corresponding author/s.
